# Influence of body size and environmental conditions on parasite assemblages of the black-spotted croaker (*Protonibea diacanthus*) (Teleostei: Sciaenidae) in northern Australia

**DOI:** 10.1017/S0031182024001008

**Published:** 2024-07

**Authors:** Megan Porter, Diane P. Barton, Joel Williams, Jo Randall, Otso Ovaskainen, David A. Crook, Shokoofeh Shamsi

**Affiliations:** 1 School of Agricultural, Environmental and Veterinary Sciences, Charles Sturt University, Wagga Wagga, NSW, Australia; 2 Gulbali Institute, Charles Sturt University, Wagga Wagga, NSW, Australia; 3 Institute for Marine and Antarctic Studies, University of Tasmania, Hobart, TAS, Australia; 4 Research Institute for the Environment and Livelihoods, Charles Darwin University, Casuarina, NT, Australia; 5 Australian Institute of Marine Science, Arafura Timor Research Facility, Casuarina, NT, Australia; 6 Department of Industry, Tourism and Trade, Northern Territory Government of Australia, Berrimah, NT, Australia; 7 Department of Biological and Environmental Science, University of Jyväskylä, Jyväskylä, Finland; 8 Organismal and Evolutionary Biology Research Programme, Faculty of Biological and Environmental Sciences, University of Helsinki, Helsinki, Finland; 9 Department of Primary Industries, Narrandera Fisheries Centre, Narrandera, NSW, Australia

**Keywords:** body size, environment, marine ecosystems, parasites, *Protonibea diacanthus*, season

## Abstract

The functioning and richness of marine systems (and biological interactions such as parasitism) are continuously influenced by a changing environment. Using hierarchical modelling of species communities (HMSC), the presence and abundance of multiple parasite species of the black-spotted croaker, *Protonibea diacanthus* (Sciaenidae), was modelled against environmental measures reflecting seasonal change. *Protonibea diacanthus* were collected in three seasons across 2019–2021 from four locations within the waters of the Northern Territory, Australia. The length of *P. diacanthus* proved to have a strong positive effect on the abundance of parasite taxa and overall parasitic assemblage of the sciaenid host. This finding introduces potential implications for parasitism in the future as fish body size responds to fishing pressure and climate changes. Of the various environmental factors measured during the tropical seasons of northern Australia, water temperature and salinity changes were shown as potential causal factors for the variance in parasite presence and abundance, with changes most influential on external parasitic organisms. As environmental factors like ocean temperature and salinity directly affect parasite–host relationships, this study suggests that parasite assemblages and the ecological functions that they perform are likely to change considerably over the coming decades in response to climate change and its proceeding effects.

## Graphical abstract

**Figure:**
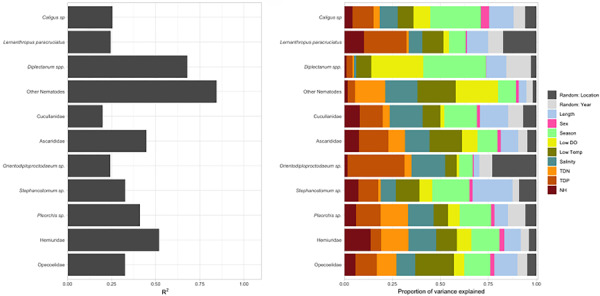

## Introduction

Fish distribution, behaviour and physiology are all affected by climatic and environmental factors, as well as biological interactions including predation and parasitism. With the increasing unpredictability of marine ecosystem functioning in evolving environmental conditions, there is a growing need to develop an understanding of the environmental impacts on both fish and their parasite communities (Lõhmus and Björklund, 2015; Poloczanska *et al*., 2016; Esbaugh, 2018). Parasitic organisms that exploit marine hosts are likely to be impacted by a changing climate, both directly through the changing ambient habitat, and indirectly *via* effects on their hosts (Lõhmus and Björklund, 2015). Many parasites require intermediate host(s) to complete their life cycle, which means that impacts may be cumulative along the life cycle (Lafferty, 2012). Parasites with life cycles that utilize free-swimming larval stages are reliant on both host availability and suitable environmental conditions for development and survival (Poulin and Leung, 2011; Lehun *et al*., 2023).

Although the environmental implications of a changing climate on marine parasites and their fish hosts are numerous, the reactions of parasites to habitat variability are not straightforward. Holmes (1990) presented a summary of the determinants of helminth community structure in marine fishes, highlighting the interactions between various biotic (fish diet and physiology) and abiotic (environmental) factors. Parasite infection rates may increase in response to minor rises in ocean temperature (Macnab and Barber, 2012; Neubert *et al*., 2016; Klimpel *et al*., 2019; Byers, 2021), whereas for other parasite taxa, infection rates decline with increasing temperature (Byers, 2021). Although difficult to predict, parasite ecology is likely to change considerably in response to climate change and its proceeding effects, and if climate impacts continue to influence marine ecosystem processes, some parasites will face suboptimal transmission conditions and/or may soon have fewer hosts available as a result of host thermal preference and host behavioural changes (Klimpel *et al*., 2019; Reynolds *et al*., 2019; Byers, 2021).

In addition to the short-term seasonal changes experienced in tropical and subtropical marine regions like northern Australia, substantial long-term environmental changes and ocean responses are expected as a result of climate change (Koenigstein *et al*., 2016). Climate models predict further warming, with ocean surfaces surrounding Australia warming at a rate less than the global average (CSIRO and Bureau of Meteorology, 2023*c*). Predictions also include increased stratification and acidification, stronger poleward currents, sea level rise and altered storm and rainfall regimes (Hobday *et al*., 2006; Poloczanska *et al*., 2007, 2016; Bindoff *et al*., 2019; Gervais *et al*., 2021). Heavy rainfall events in Australia have increased in intensity (CSIRO and Bureau of Meteorology, 2023*a*), with regions surrounding the equator like the north of Australia generally experiencing wetter years (CSIRO and Bureau of Meteorology, 2023*b*). In addition to rainfall changes, the average sea-surface temperature of Australia has also increased by more than 1°C since 1900, including eight of the ten warmest years since 2010 (CSIRO and Bureau of Meteorology, 2023*a*).

Climate change studies predict that ocean warming will trigger a polar shift in the distribution of marine organisms, leading to decline in fish species richness in tropical waters, along with changes in sea-surface temperatures, salinity levels and the levels of dissolved oxygen (Barange and Perry, 2009; Bindoff *et al*., 2019; Yang *et al*., 2023). In addition to population movements, the physiology of several marine fish species has been strongly influenced by temperature-driven changes in recruitment and somatic growth, with overall reductions in size recorded in reproductively active stock (Sheridan and Bickford, 2011; Lindmark *et al*., 2022). The black-spotted croaker *Protonibea diacanthus* (Teleostei: Sciaenidae) is a large marine fish species of considerable value to recreational, traditional and commercial fishing sectors of northern Australia (Phelan *et al*., 2008; Saunders *et al*., 2021), and is one marine species likely to be impacted by ocean warming, with distribution shifts reported in several marine fish species (Jacups, 2010; Poloczanska *et al*., 2013; Zhang *et al*., 2019; Gervais *et al*., 2021). *Protonibea diacanthus* is distributed in the wet–dry tropics of northern Australia, occurring throughout the Indo-west Pacific region, including Papua New Guinea, and reaching from the Persian Gulf to Japan (Bray, 2022; Randall *et al*., 2023). Inhabiting the wet–dry tropics, *P. diacanthus* is often exposed to minor environmental changes as a result of the tropical weather conditions and seasonal changes, including changes to water quality, composition and movement. The monsoonal wet season in northern Australia brings significant rainfall over the months from December to March, typically resulting in elevated freshwater run-off from river systems into nearshore estuarine and coastal habitats. The combination of freshwater run-off, sediment mixing and tidal flows during the wet season often leads to significant changes in water temperature, salinity and chemical composition of coastal marine waters (Anderson *et al*., 2011). The dry season, typically between April and August, is a period of negligible rainfall and low run-off of freshwater into the environment. The transition period from dry to wet, referred to as the ‘build-up’ season, occurs between September and November, and is associated with rises in temperature and humidity, and increasing rainfall mainly from irregular, non-monsoonal storms (Porter *et al*., 2023*b*).

As natural components of ecological systems, parasitic organisms are expected to react to environmental changes and the subsequent behavioural changes, health impacts and movement patterns of their hosts (Byers, 2021). With previous studies highlighting the richness of parasite infection in *P. diacanthus* (Porter *et al*., 2023a, 2023*b*, 2023*c*), there is a need to understand the potential climate-induced pressures that this major parasite–host system may face. This study investigated the parasites occurring in *P. diacanthus* populations off the northern coast of Australia and modelled the presence and abundance of multiple parasite taxa relative to environmental variables. The results of this study are discussed with respect to the impacts of environmental change on parasites, and how these changes might manifest as patterns of parasite prevalence, abundance and diversity. In understanding how parasites respond to seasonal variability, this study aims to improve the capacity to predict how parasites (and their impacts on hosts) will respond to environmental variation in a changing climate.

## Materials and methods

### Fish and parasite collection

**Figure 1.**
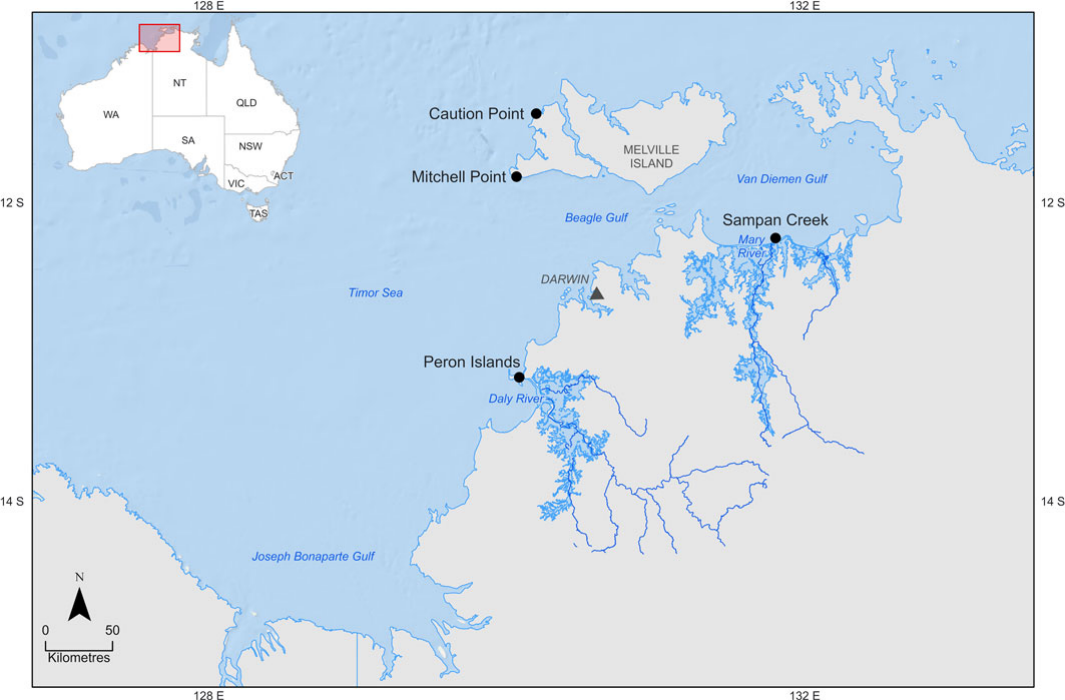
Collection sites of *Protonibea diacanthus* from northern Australia. Map sourced from the Office of Research Services and Graduate Studies, Spatial Data Analysis Network (SPAN), Charles Sturt University, Wagga Wagga, Australia.

The fish and parasites included in this study are as described in Porter *et al*. (2023*b*). In brief, 176 *P. diacanthus* were collected from four coastal locations off the Northern Territory coast (Fig. 1). The fish were sampled from two nearshore locations in proximity to the mouths of the Daly River and the Mary River (Peron Islands and Sampan Creek, respectively), and two offshore locations of the Tiwi Islands (Caution Point and Mitchell Point). The sites chosen allowed comparison between nearshore and offshore sites, with nearshore locations receiving freshwater outflow in the wet season, and those offshore more reflective of oceanic conditions with no freshwater outflow nearby. Fish were captured at these sites during three seasonal sampling periods in 2019–2021 including: build-up (October–November 2019 and 2020), late-wet (February–March 2020 and 2021) and mid-dry (June–July 2019 and 2020), with monsoonal wet season rainfall occurring from December to March, and the negligible rainfall during the dry season between April and August. Parasites were collected from the gills and the gastrointestinal systems of each fish and identified to as low a taxonomic level as possible.

### Environmental data

At the time of capture of fish, environmental data were collected at the water surface and at the depth of capture, which varied between 3 and 30 m, depending on the location: Sampan Creek 3‒8 m, Caution Point 10‒15 m, Peron Islands 15‒25 m and Mitchell Point 20‒30 m. Environmental variables measured were: dissolved oxygen, water temperature, salinity, ammonia, total dissolved nitrogen and total dissolved phosphorus. Given *P. diacanthus* is considered a demersal species (Bray, 2022), the measures of dissolved oxygen and water temperature from the lower part of the water column were used in the analyses. Ammonia, total dissolved nitrogen, and total dissolved phosphorus were examined as potential indicators of freshwater outflow at the sampling sites.

### Data analysis

Summary statistics for the parasite and environmental data were compiled for each location by season of collection (Table 1). Due to low sample sizes, data across the two years were grouped by season of collection. For each of the parasite taxa, mean abundance (the total number of individuals of a particular parasite per sample divided by the total number of hosts examined, including uninfected hosts), and prevalence (the number of hosts infected with a particular parasite divided by the number of hosts examined, expressed as a percentage) were calculated (Bush *et al*., 1997). The mean values of each environmental variable were also calculated and compiled for each location by season of collection (Table 2).

**Table 1.** Prevalence and mean abundance of parasites from *Protonibea diacanthus*, across seasons (Mid-dry, Build-up and Late-wet), and sites (Caution Point, Mitchell Point, Peron Islands, Sampan Creek)

	Mid-dry								Build-up								Late-wet							
	Caution Point		Mitchell Point		Peron Islands		Sampan Creek		Caution Point		Mitchell Point		Peron Islands		Sampan Creek		Caution Point		Mitchell Point		Peron Islands		Sampan Creek	
Number of fish	19		10		17		18		7		10		10		20		16		20		20		9	
Mean length (cm)	98.6		98.5		108.3		112.6		84.9		101.0		108.7		112.4		107.8		92.8		85.1		123.6	
Parasite taxa	*P*	*A_M_*	*P*	*A_M_*	*P*	*A_M_*	*P*	*A_M_*	*P*	*A_M_*	*P*	*A_M_*	*P*	*A_M_*	*P*	*A_M_*	*P*	*A_M_*	*P*	*A_M_*	*P*	*A_M_*	*P*	*A_M_*
**Copepoda** *Caligus* sp.	10.5	0.1	0.0	0.0	70.6	1.6	22.2	0.4	28.6	0.3	0.0	0.0	20.0	0.2	5.0	0.1	0.0	0.0	0.0	0.0	25.0	0.5	0.0	0.0
*Lernanthropus* sp.	78.9	2.1	100.0	6.7	52.9	2.9	83.3	3.9	71.4	3.1	100.0	5.7	100.0	4.2	95.0	12.9	93.8	7.9	70.0	2.6	90.0	2.6	100.0	8.4
**Monogenea** *Diplectanum* spp.	100.0	834.1	100.0	1223.0	100.0	1204.2	100.0	1058.4	100.0	1262.7	100.0	2498.0	100.0	39.6	100.0	195.2	100.0	2658.8	100.0	829.0	100.0	544.0	100.0	3293.3
**Nematoda** Cucullanidae	100.0	21.9	90.0	24.4	94.1	18.5	94.4	18.9	100.0	18.9	100.0	11.8	100.0	10.7	85.0	24.0	93.8	56.4	100.0	13.0	100.0	10.1	100.0	57.9
Ascarididae	5.3	0.1	0.0	0.0	0.0	0.0	27.8	0.4	0.0	0.0	0.0	0.0	10.0	0.2	15.0	0.3	0.0	0.0	0.0	0.0	0.0	0.0	0.0	0.0
Other Nematodes	26.3	0.3	10.0	0.1	23.5	0.4	11.1	0.1	0.0	0.0	10.0	0.3	10.0	0.1	20.0	0.3	0.0	0.0	0.0	0.0	10.0	0.3	0.0	0.0
**Digenea** *Orientodiploproctodaeum* sp.	100.0	21.5	100.0	39.1	100.0	49.8	83.3	6.9	100.0	19.4	100.0	38.3	100.0	26.4	85.0	13.1	100.0	32.3	90.0	13.8	100.0	23.8	88.9	19.3
*Stephanostomum* sp.	89.5	14.8	100.0	33.3	82.4	8.2	88.9	30.1	100.0	12.9	100.0	21.0	70.0	4.9	85.0	10.2	81.3	30.9	85.0	11.3	65.0	3.4	100.0	57.0
*Pleorchis* sp.	10.5	0.1	80.0	5.3	5.9	0.1	0.0	0.0	0.0	0.0	30.0	0.4	0.0	0.0	0.0	0.0	12.5	0.3	25.0	0.5	10.0	0.2	0.0	0.0
Hemiuridae	0.0	0.0	0.0	0.0	17.6	0.5	16.7	0.2	14.3	0.1	0.0	0.0	0.0	0.0	0.0	0.0	6.3	0.2	10.0	0.2	30.0	0.5	11.1	0.8
Opecoelidae	5.3	0.1	0.0	0.0	35.3	0.8	27.8	0.4	0.0	0.0	10.0	0.2	0.0	0.0	5.0	0.1	0.0	0.0	0.0	0.0	25.0	0.6	0.0	0.0

Prevalence, expressed as a percentage, is the number of hosts infected with a particular parasite divided by the number of hosts examined. Mean Abundance is expressed as the total number of individuals of a particular parasite per sample divided by the total number of hosts examined, including uninfected hosts.

**Table 2.** Mean values of environmental measures taken for seasons and sites

	Mid-dry				Build-up				Late-wet			
	Caution Point	Mitchell Point	Peron Islands	Sampan Creek	Caution Point	Mitchell Point	Peron Islands	Sampan Creek	Caution Point	Mitchell Point	Peron Islands	Sampan Creek
Dissolved oxygen (mg L^−1^)	6.4	6.2	6.5	6.8	7.5	7.8	5.6	6.0	6.0	6.0	5.8	6.6
Water temperature (°C)	26.3	26.9	23.8	25.0	29.9	29.6	31.2	30.9	29.9	29.9	30.6	30.2
Salinity (from >2 m)	35.4	36.0	35.5	35.4	35.1	35.1	36.2	36.7	34.6	35.2	32.6	29.6
Ammonia	0.5	0.5	1.1	0.6	1.0	0.3	2.2	0.2	0.3	0.8	1.6	0.2
Total dissolved nitrogen	4.4	10.2	10.2	6.5	13.1	5.6	5.5	4.4	7.1	10.2	9.6	11.0
Total dissolved phosphorus	0.3	0.3	0.3	0.4	0.3	0.4	0.2	0.3	0.3	0.4	0.5	0.7

For the statistical analysis, the data were analysed using a joint species distribution model called hierarchical modelling of species communities (HMSC; Ovaskainen *et al*., 2017; Ovaskainen and Abrego, 2020). The response data used in the model comprise the abundance of parasite taxa from the fish host. For the analyses, the individual fish were used as sampling units and the count of each of the 11 parasite taxa were used as the response variable. To account for zero-inflation for some of the parasite taxa, a hurdle model was applied, i.e. one model for presence–absence of the taxa, and another model for the abundance of taxa conditional on presence. Probit regression was applied in the presence–absence model, and linear regression for log transformed count data in the abundance conditional on presence model. The count data were transformed by declaring zeros as missing data, log-transforming, and then scaling the data to zero mean and unit variance within each taxa. Fish collection year and location were included as random effects, and fish collection season, fish length, fish sex and six environmental variables: dissolved oxygen, water temperature, salinity, ammonia, total dissolved nitrogen and total dissolved phosphorus, were included as fixed effects. As species traits, the categorical variable of internal or external parasites was also applied.

Both models were fitted using the HMSC package from R (Tikhonov *et al*., 2020) assuming the default prior distributions (Ovaskainen and Abrego, 2020). The posterior distribution was sampled using four Markov chain Monte Carlo (MCMC) chains. Each chain consisted of 37 500 iterations, of which 12 500 iterations were removed as burn-in and the remaining thinned by 100 to result in 250 posterior samples per chain, so there were 1000 posterior samples in total. The MCMC convergence diagnostics were examined through the potential scale reduction factors of the model parameters (Gelman and Rubin, 1992). Both the explanatory powers and the predictive powers were examined for each of the models, with measures of the species-specific AUC and Tjur's *R*^2^ (similar to *R*^2^, Tjur, 2009) examined for the presence–absence model (Pearce and Ferrier, 2000), and *R*^2^ measured for the abundance conditional on the presence model. To compute the explanatory power, model predictions were made based on the models being fitted to all of the data. The predictive power was computed by performing a five-fold cross validation, in which the sampling units were assigned randomly to five folds, and predictions for each fold were based on the model that was fitted to the data on the remaining four folds.

To quantify the drivers of parasite taxa richness and abundance, the explained variation was partitioned among the fixed and random effects included in the model. To examine associations between parasite taxa and environmental variables, parasite responses to the explanatory variables were measured, counting the proportion of parasites showing positive or negative associations with at least 95% posterior probability.

## Results

All 176 fish were infected with at least one parasite and a total of 11 parasite taxa were identified from the gills and the gastrointestinal system for use in the analysis (Table 1). External parasites included copepods, *Lernanthropus paracruciatus* Boxshall, Bernot, Barton, Diggles, Yong, Atkinson-Coyle & Hutson, 2020 (see Boxshall *et al*., 2020) and *Caligus* sp., and two species of monogeneans, *Diplectanum timorcanthus* Porter, Barton, Francis & Shamsi, 2023 and *Diplectanum diacanthi* Porter, Barton, Francis & Shamsi, 2023 (see Porter *et al*., 2023a), combined as *Diplectanum* spp. due to the difficulty in distinction between the species at time of dissection. Of the internal parasites included in the analysis there were adult nematodes belonging to the families Cucullanidae and Ascarididae, and a further group of nematodes not yet identified but classified as ‘Other Nematodes’ given their similarities in morphological characteristics and general ecology. Adult digenean trematodes *Orientodiploproctodaeum* sp., *Stephanostomum* sp., *Pleorchis* sp., and representatives from the families Hemiuridae and Opecoelidae were also included.

The environmental variables differed between locations based on seasons (Table 2). The water temperature of the inshore locations of Peron Islands and Sampan Creek differed from that of the offshore locations, being lower in the mid-dry, but higher in the build-up and late-wet. Salinity at the inshore locations in the build-up was markedly lower in the late-wet season, indicating a freshwater influence at these sites. Levels of ammonia did not exhibit a clear seasonal pattern. Between seasons only, levels of nitrogen and phosphorus were higher in the late-wet season than in the mid-dry and build-up seasons.

The MCMC convergence diagnostics of the HMSC models were satisfactory, meaning that the models were adequately fitted to the data (Ovaskainen and Abrego, 2020). Namely, the potential scale reduction factors for the *β*-parameters were on average 1.002 (0.998‒1.013) for the presence–absence model and 1.002 (0.997‒1.009) for the abundance conditional on presence model. The presence–absence models showed a good fit to the data, the mean Tjur *R*^2^ (AUC) reported as 0.186 (0.861) for explanatory power, and 0.108 (0.693) predictive power. The abundance conditional on the presence model showed satisfactory model fit, with the mean *R*^2^ value being 0.407 for explanatory power and 0.095 for predictive power. *Diplectanum* spp. (prevalence 100%) and Cucullanidae (prevalence 96%), were present in (almost) all samples and therefore not considered informative for the presence–absence model.

**Figure 2.**
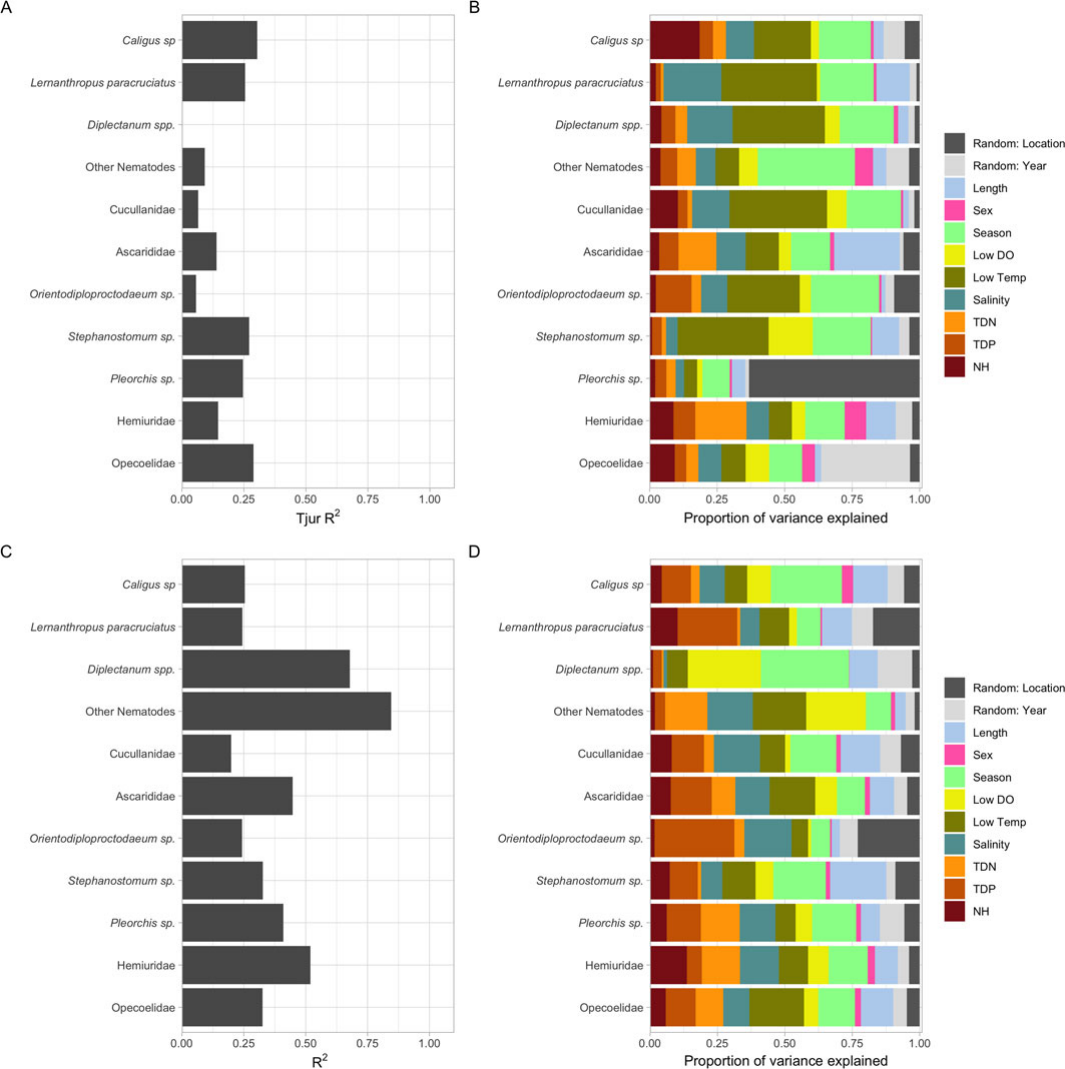
(A) Plot of explanatory power for the presence–absence model highlighted through the Tjur *R*^2^ values of the parasite species, (B) plot of variance partition over the explanatory variables in the presence–absence model, showing the proportion of variance explained by both the random effects and the fixed effects for the parasite species, (C) plot of explanatory power of the abundance conditional on presence model highlighted through the *R*^2^ values of the parasite species, (D) plot of variance partition over the explanatory variables in the abundance conditional on presence model, showing the proportion of variance explained by both the random effects and the fixed effects for the parasite species. NB: Abbreviations for environmental variables have been used and include dissolved oxygen as (LowDO), water temperature as (LowTemp), ammonia as (NH), total dissolved nitrogen as (TDN) and total dissolved phosphorus as (TDP).

For the presence–absence model the explanatory power for each species was low (Fig. 2A), suggesting the variables use in the model only explain a small proportion of the variance and therefore the distribution of parasite taxa is more by random chance than by the environment. Variance partition over the explanatory variables included in the presence–absence model showed that the proportion of the fixed effects of water temperature and season explained between 20 and 40% of variance for most parasites, with salinity explaining the next highest proportion between 10 and 20% (Fig. 2B). The explanatory power of the abundance conditional on the presence model (Fig. 2C) showed that five parasite taxa explained greater than 40% of variance, with the *Diplectanum* spp. and ‘Other Nematodes’ explaining the highest proportions of over 60% of variance. Out of the explained variance for the abundance conditional on the presence model, the fixed effects that explained most of the variance included salinity, water temperature, length and season (Fig. 2D). Although still not recording the highest proportion of explained variance, the fixed effects of total dissolved nitrogen and total dissolved phosphorus were much more correlated with most parasite taxa in the abundance conditional on the presence model, when compared with the presence–absence model. For the abundance of external parasites, season explained the most variance for both *Caligus* sp. and *Diplectanum* spp. (Fig. 2D).

**Figure 3.**
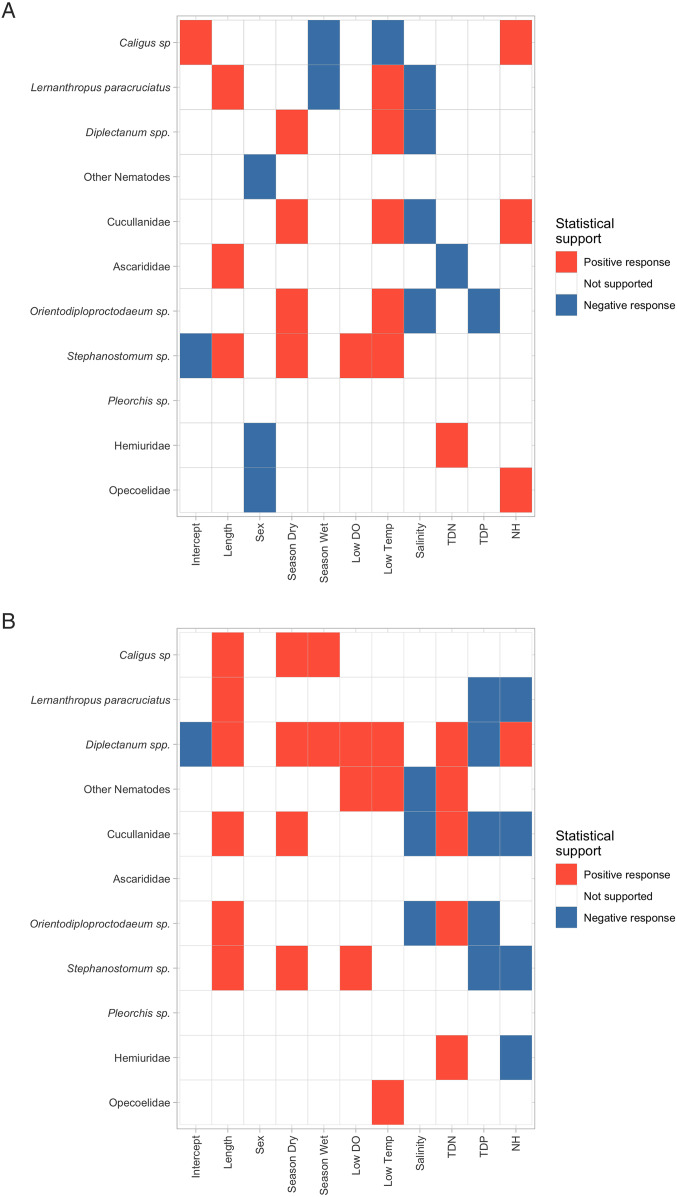
(A) Beta plot of the species responses to the explanatory variables in the presence–absence model with at least 95% posterior probability, (B) Beta plot of the species responses to the explanatory variables in the abundance conditional on presence model with at least 95% posterior probability. NB: In 3A and 3B, the red and blue colours indicate those parasite taxa–environmental variable pairs with at least 0.95 support for either a positive or negative association, respectively. If there is no colour, the parasite taxa presence/absence or abundance conditional on presence is deemed to not have an association with the environmental variable, i.e. the taxa are neither positively nor negatively influenced by the environmental variable. Abbreviations of environmental variables are as those described in Fig. 2.

The beta plot of the presence–absence model showed *L. paracruciatus* and *Caligus* sp. to have a negative response to the late-wet season (Fig. 3A). In the beta plot of the presence–absence model, *L. paracruciatus* and the two most prevalent internal parasites (*Orientodiploproctodaeum* sp. and *Stephanostomum* sp.) showed increasing occurrence probability with increasing water temperature. Interestingly, all but *Stephanostomum* sp. had a negative association with salinity (Fig. 3A).

The beta plot of the abundance conditional on the presence model demonstrated that more than half of all parasite taxa are significantly more abundant as fish length increases (Fig. 3B). *Diplectanum* spp. abundance has a positive association with many environmental variables including both the mid-dry and the late-wet seasons (relative to the build-up season), dissolved oxygen and temperature, ammonia and total dissolved nitrogen (Fig. 3B). The abundance of both *L. paracruciatus* and *Diplectanum* spp. had a negative association with total dissolved phosphorus. In all, five parasite taxa were negatively associated with total dissolved phosphorus (Fig. 3B).

**Figure 4.**
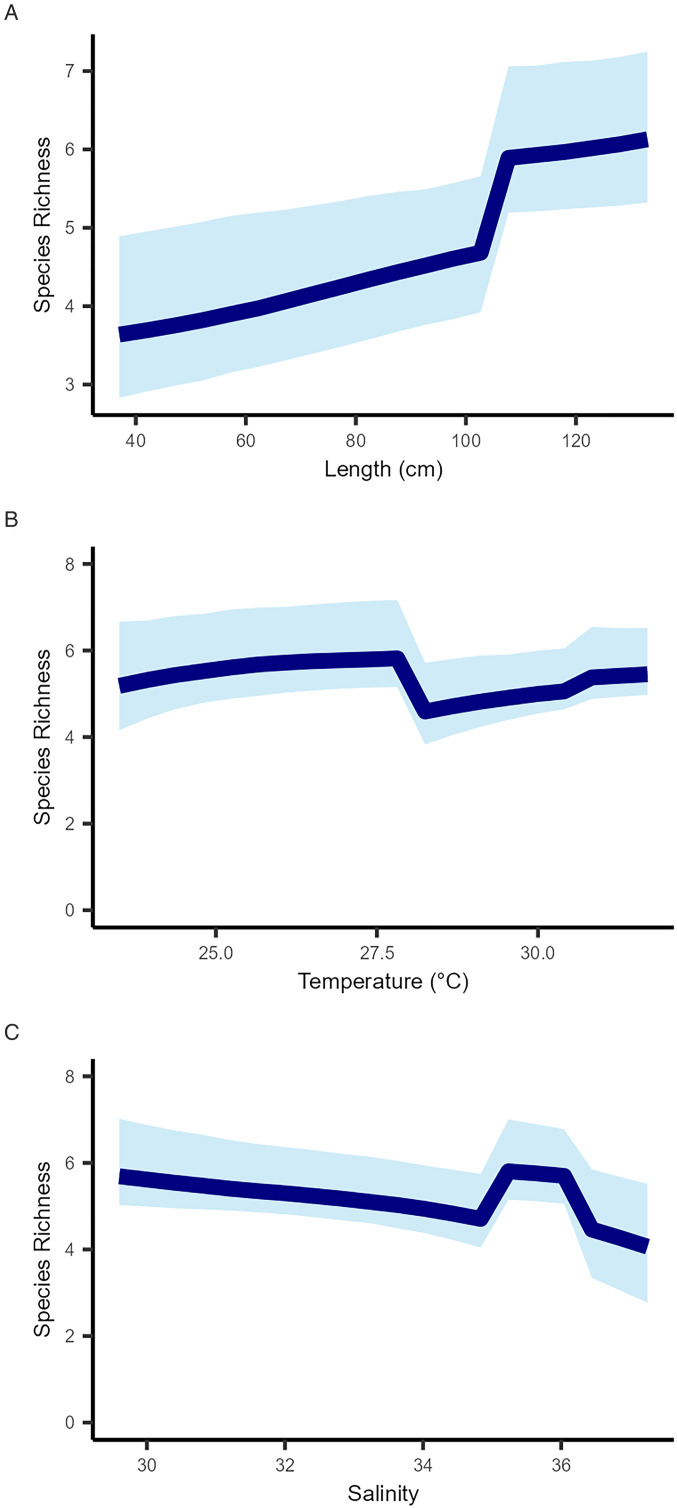
Gradient plots of the relationships between: (A) Parasite species richness and fish length from all samples (cm), (B) Parasite species richness and water temperature (°C), (C) Parasite species richness and salinity. NB: Parasite species richness is a reflection of the number of different parasite species that are known to infect a particular host (or host population).

The presence–absence model predicts that parasite richness is positively correlated with fish length (Fig. 4A). Parasite species richness was relatively consistent as temperature changes, however, there was a drop in richness at temperatures between 27 and 28°C (Fig. 4B). This drop occurred at only the one location and season at which this temperature was recorded (Table 2), and when four parasite taxa were not present (Table 1). Species richness was highest when salinity levels were low, with a drop recorded when salinity exceeded 36‰ (Fig. 4C).

## Discussion

The presence and abundance of parasites were significantly influenced by the body size of *P. diacanthus*, with the effect of body size on parasites proving the strongest factor across the seasons. Positive associations between body size and parasite presence and abundance have been reported in numerous previous studies and stock analyses of marine fishes (Thoney, 1993; Jerry *et al*., 2013; Welch *et al*., 2015; Barton *et al*., 2018; Taillebois *et al*., 2017; Taillebois *et al*., 2021; Kouadio *et al*., 2023). Studies confirm a general link between host size and parasite richness, however there is an obvious need to consider that this complex association is far from a simple linear relationship (Poulin *et al*., 2011). Host size corresponds closely to the size of habitat available to parasitic fauna and is also correlated with host life span, meaning that larger hosts offer a larger habitat patch and feature longer-lived habitats, therefore harbouring richer parasite faunas (Poulin and Morand, 2004; Poulin *et al*., 2011). This is justified with the marked increase in parasite species richness shown as the host size of *P. diacanthus* exceeds 100 cm. Food selection amongst fishes is potentially influenced by both temperature and body size (Coghlan *et al*., 2024), and as fish mature, changes in feeding modes and diet composition are common, with *P. diacanthus* for example known to transition from small invertebrates such as crabs and prawns, to larger prey items like fish (Barton, 2018). This dietary change would certainly influence the parasitic fauna of the fish host, and given this occurs as a result of maturation, can further support the association between fish host size and parasite species richness. Studies also suggest that greater abundances of parasites are found in offshore fish communities when compared with their juvenile counterparts nearshore, suggesting that certain parasites are in fact accumulated overtime, or that there is a potentially greater number of infected intermediate hosts offshore (Thoney, 1993).

Changes in body size of fish in response to ocean warming are often attributed to the temperature-size rule (TSR), with ectotherms reared at warmer temperatures developing faster but reaching smaller ultimate body sizes (Ohlberger, 2013; Lindmark *et al*., 2022). This finding has correlated with observations for several commercially exploited marine fish species (Olsen *et al*., 2004; Andersen *et al*., 2007; Baudron *et al*., 2014; Rijn *et al*., 2017; Ikpewe *et al*., 2021; Wootton *et al*., 2022), with negative size shifts impeding the maintenance and recovery of exploited marine fish populations whose larger individuals are typically the most fecund (Genner *et al*., 2010). Given that reductions in fish size have been observed as ecological responses to increasing ocean temperatures (Sheridan and Bickford, 2011; Audzijonyte *et al*., 2020), it appears likely that climate change will have significant impacts on associations between parasites and their hosts in the future. Considering the important role of parasites in marine food webs and ecosystem processes (Timi and Poulin, 2020; Porter *et al*., 2023*b*, 2023*c*), the results highlight the need to improve understanding of the secondary effects of climate change on host parasite dynamics, and the potential future implications to marine ecosystems (Byers, 2021).

To assess the potential effects of climate change on parasite communities, it is important to consider the broad marine ecosystem impacts and adaptations in host biology that also impact parasites. The present study demonstrates that environmental variables were strongly associated with parasite richness and abundance in *P. diacanthus*. The variance in parasite presence and abundance can be partially explained by seasonal changes in environmental conditions, which include water temperature, salinity levels, and nitrogen and phosphorus. The short-term seasonal environmental factors varied between locations and seasons in this study, with consistently warmer water temperatures and associated lower levels of dissolved oxygen during the late-wet season and at Peron Islands during the build-up season. Oxygenation stress has been reported to increase parasite prevalence and infection success in vulnerable hosts with effects seen in both fish metabolic function and physiology (Mikheev *et al*., 2014; Poloczanska *et al*., 2016; Byers, 2021; Samaras *et al*., 2023). Rising global temperatures lead to decreased oxygen solubility in water (Breitburg *et al*., 2018), and thus it is expected that the wet season of northern Australia will produce lower water oxygen levels in association with warmer ocean temperatures in the future. In addition to oxygen negatively covarying with temperature, oxygen declines in estuaries and nearshore marine ecosystems have also been caused by increased loadings of nutrients (Breitburg *et al*., 2018). Nutrient levels of nitrogen and phosphorus were highest at the nearshore sites during the late-wet season when these areas of northern Australia are often inundated with stormwater and run-off from river systems, floodplains and agricultural land (Przeslawski *et al*., 2011). The levels of phosphorus in this study are related to water column turbidity during seasonal outflows (Kämäri *et al*., 2020), and it is possible that climate change may bring more frequent, high levels of outflow. These run-off events and changes in water composition (including during tidal movements) cause critical disturbance to the ecosystem, influencing levels of energy and nutrients, and affecting the composition of benthic organisms (Anderson *et al*., 2011).

It is important to recognize that different parasite species respond differently to environmental changes, and as to whether parasite infections increase or decrease as a result of climate changes will often come down to the individual species at hand (Mackenzie, 1999). External parasites, such as the copepods and monogeneans from this study, exhibit very different morphological characteristics such as the nature of their tegument and their size, both of which are highly influential on the level of sensitivity that these organisms reflect when faced with the surrounding changes in factors like water temperature and salinity (Möller, 1978). The two most prevalent external parasites of this study, *L. paracruciatus* and *Diplectanum* spp., demonstrated a negative association with salinity. *Lernanthropus paracruciatus* was also significantly more likely to be present during warmer conditions, whereas *Caligus* sp. was more likely to be absent during the late-wet season when the water temperature was at its warmest. *Caligus* sp. did not show a significant association with salinity, indicating that the parasites may not be as influenced by changes in salinity as *L. paracruciatus* and *Diplectanum* spp. Previous studies have similarly described changes in external parasite abundance in warmer, saline conditions, with reduced presence at low salinities (Bricknell *et al*., 2006; Callaway *et al*., 2012; Byers, 2021).

The development and diversity of some parasite species depends on the presence of suitable intermediate hosts, in combination with other abiotic and biotic factors (Klimpel *et al*., 2019). Internal parasites, such as the digeneans and nematodes in the present study, may be buffered from direct environmental variation, but may be affected by the parasite's reliance on an earlier host (Neubert *et al*., 2016; Byers, 2021). Any environmental influence on the internal parasite–host system in the present study may not be reflective of seasonal cycles but instead of a ‘lag effect’ due to the indirect impacts on internal parasites or their dependence on intermediate hosts. For example, the significantly lower temperature during the preceding mid-dry season can affect parasite infectivity, longevity, and survival to transmission of free-living stages of internal parasites (Pietrock and Marcogliese, 2003; Lõhmus and Björklund, 2015) and may also lower the activity of both intermediate hosts (molluscs, small fishes and crustacea) and *P. diacanthus* (Lõhmus and Björklund, 2015). This can ultimately result in lower transmission of larval digeneans (whose capsule is often fragile and delicate) (Pietrock and Marcogliese, 2003) between hosts, or from vegetation, and therefore lower abundance of adult stages in *P. diacanthus* during the build-up. Thus, it is important for future research to understand whether changes in the dynamics of parasite assemblages' is a cumulative effect over many seasons or whether changes are truly reflective of what occurs in each individual season. On the temporal scale of global climate change, overall influences on the internal parasite–host systems may accumulate and present somewhat differently to the influences caused by short-term seasonal environmental variation.

This study has shown that seasonal environmental variation has an impact on the abundance and distribution of parasites. The effects of short-term seasonal environmental variation and fish host size, provides a window into the potential future of parasite–host systems when exposed to longer term climate variation. As ocean temperatures continue to warm and the impacts of climate change play out across broad-scale ecosystem processes, changes in trophic structure, energy flow and nutrient dynamics are to be expected. Given the varying ecology of parasitic organisms, individual responses to climate changes are difficult to predict. Nonetheless, positive correlations between *P. diacanthus* length and parasite assemblages – and the complex associations with environmental variables – suggest that fundamental shifts in parasite–host dynamics for marine fishes are likely under a changing climate.

## Supporting information

Porter et al. supplementary material Porter et al. supplementary material

## Data Availability

All data produced for this study are provided in the manuscript.

## Supplementary material

The supplementary material for this article can be found at https://doi.org/10.1017/S0031182024001008.
